# Total Flavonoids from *Rosa laevigata* Michx Fruit Ameliorates Hepatic Ischemia/Reperfusion Injury through Inhibition of Oxidative Stress and Inflammation in Rats

**DOI:** 10.3390/nu8070418

**Published:** 2016-07-08

**Authors:** Xufeng Tao, Xiance Sun, Lina Xu, Lianhong Yin, Xu Han, Yan Qi, Youwei Xu, Yanyan Zhao, Changyuan Wang, Jinyong Peng

**Affiliations:** 1 College of Pharmacy, Dalian Medical University, Western 9 Lvshunnan Road, Dalian 116044, China; taoxufengdalian@163.com (X.T.); Linaxu_632@126.com (L.X.); Lianhongyin_1980@163.com (L.Y.); Xuhan2002zs@163.com (X.H.); Yanqi_1976@163.com (Y.Q.); Youweixu_1964@163.com (Y.X.); Yanyanzhao_2009@126.com (Y.Z.); yuhao.1988517zs@163.com (C.W.); 2 Department of Occupational and Environmental of Health, Dalian Medical University, No. 9 Western Section of Lushun South Road, Dalian 116044, China; qimengdy2020@163.com

**Keywords:** hepatic ischemia/reperfusion, inflammation, oxidative stress, *Rosa laevigata* Michx fruit, total flavonoids

## Abstract

The effects of total flavonoids (TFs) from *Rosa laevigata* Michx fruit against liver damage and cerebral ischemia/reperfusion (I/R) injury have been reported, but its action on hepatic I/R injury remains unknown. In this work, the effects and possible mechanisms of TFs against hepatic I/R injury were examined using a 70% partial hepatic warm ischemia rat model. The results demonstrated TFs decreased serum aspartate transaminase (AST), alanine aminotransferase (ALT), myeloperoxidase (MPO), and lactate dehydrogenase (LDH) activities, improved liver histopathology and ultrastructure through hematoxylin-eosin (HE) staining and electron microscope observation. In addition, TFs significantly decreased malondialdehyde (MDA) and increased the levels of superoxide dismutase (SOD) and glutathione peroxidase (GSH-Px), which indicated that TFs alleviated oxidative stress caused by I/R injury. RT-PCR results proved that TFs downregulated the gene levels of inflammatory factors including interleukin-1 beta (IL-1β), interleukin-1 (IL-6), and tumor necrosis factor alpha (TNF-α). Further research indicated that TF-induced hepatoprotection was completed through inhibiting TLR4/MyD88 and activating Sirt1/Nrf2 signaling pathways. Blockade of the TLR4 pathway by TFs inhibited NF-κB and AP-1 transcriptional activities and inflammatory reaction. Activation of Sirt1/Nrf2 pathway by TFs increased the protein levels of HO-1 and GST to improve oxidative stress. Collectively, these findingsconfirmed the potent effects of TFs against hepatic I/R injury, which should be developed as a candidate for the prevention of this disease.

## 1. Introduction

Ischemia/reperfusion (I/R) injury is a pathologic process occurring in the organs that suffer temporary blood flow deprivation (ischemia) and restoration (reperfusion) [1]. Clinically, hepatic I/R injury always occurs in a number of settings, including hepatic transplantation, hepatic resection, and hemorrhagic shock, which can lead to higher incidences of acute and chronic organ failure [2]. Patients who suffer from hepatic I/R are exposed to enormous pain and financial burdens [3]. However, no ideal drugs show good efficiency to cure hepatic I/R injury at the clinical level [4]. Therefore, it is urgent to develop new and effective therapies for the treatment of hepatic I/R injury.

Many basic and clinical experiments have demonstrated that hepatic I/R can induce direct cellular insult and delayed dysfunction, as well as the injury resulting from activating multiple oxidative stress and inflammatory pathways [5,6,7]. Sirtuin 1 (Sirt1) is a nicotinamide adenine dinucleotide (NAD^+^)-dependent nuclear class III histone deacetylase that participates in theregulation of metabolic and oxidative stress [8]. Briefly, a transcription factor-nuclear erythroid factor 2-related factorn2 (Nrf2) is anchored in the cytoplasm where it binds to Kelch-like ECH-associated protein 1 (Keap1) under normal circumstances [9]. However, Nrf2 translocates into the nucleus and then activates its target genes through an antioxidant-response element (ARE) when Sirt1 triggers the separation of Nrf2 and Keap1 [10]. Among the target genes of Nrf2, heme oxygenase-1 (HO-1), and glutathione-*S*-transferase (GST) are two anti-oxidative stress representatives [11]. HO-1 can catalyze heme metabolism to eliminate free radicals [12]. GST, one xenobiotic-metabolizing enzyme, can catalyze the nucleophilic attack of reactive oxygen species (ROS) and help to detoxify [13]. Accordingly, Sirt1/Nrf2 signaling can activate some antioxidant enzymes to improve the cellular redox state.

Furthermore, inflammatory response is well known to concern the activation of congenital immunity through binding toll-like receptor 4 (TLR4) with endogenous ligands in the absence of pathogens [14]. Recent reports have shown that activated TLR4 can trigger TNF receptor-associated factor 6 (TRAF6) by its adaptor protein myeloid differentiation primary response gene (88) (MyD88) [15]. Then, TRAF6 increases nuclear factor kappa B (NF-κB) translocation and c-Jun *N*-terminal kinase (JNK) phosphorylation that subsequently stimulates activator protein 1 (AP-1) transcription [16]. Ultimately, these molecules cause the release of a large number of inflammation cytokines including interleukin-1 beta (IL-1β), interleukin-1 (IL-6), and tumor necrosis factor alpha (TNF-α) after warm hepatic I/R [17]. Thus, many studies have focused on regulation of immune function to alleviate hepatic I/R injury.

*Rosa laevigata* Michx fruit has been used in China for a long history to treat chronic cough, arterial sclerosis, menstrual irregularities, and urinary incontinence [18,19], which mainly contains polysaccharose, flavonoids, and saponins [20,21]. The crude extract of total flavonoids (TFs) from it mainly contains flavones and flavonols, including quercetin, kaempferide, apigenin, and isorhamnetin [22,23]. Our previous investigations have demonstrated that TFs have hepatoprotective effects against high-fat diet and carbon tetrachloride-induced liver damage [24,25]. We also indicated that TFs have potent effects against cerebral I/R injury [26]. Nevertheless, to the best of our knowledge, no work has been investigated to report the actions of TFs against hepatic I/R injury.

Thus, the aim of this paper was to investigate the effects and possible mechanisms of TFs from *R. laevigata* Michx fruit against liver I/R damage.

## 2. Material and Methods

### 2.1. Chemicals and Materials

D101 macroporous resin was purchased from the chemical plant of Nankai University (Tianjin, China). Aspartate transaminase (AST, Code No. C010-1), alanine aminotransferase (ALT, Code No. C009-1), myeloperoxidase (MPO, Code No. A044), lactate dehydrogenase (LDH, Code No. A020-1), malondialdehyde (MDA, Code No. A003-1), superoxide dismutase (SOD, Code No. A001-1), and glutathione (GSH, No. A005) kits were obtained from Nanjing Jiancheng Institute of Biotechnology (Nanjing, China). Hematoxylin (Code No. ZLI9606), eosin (Code No. ZLI9612), and diaminobenzidine (DAB, Code No. ZLI9632) staining kits were purchased from Zhongshan Golden Bridge Biotechnology (Beijing, China). Tissue Protein Extraction Kit (Code No. KGP2100) and Nuclear and Cytoplasmic Protein Extraction kit (Code No. KGP150) were obtained from KEYGEN Biotech. Co., Ltd. (Nanjing, China). Bicinchoninic acid Protein Assay Kit (BCA, Code No. P0012S) was purchased from Beyotime Institute of Biotechnology (Shanghai, China). RNAiso Plus (Code No. 9109), PrimeScript™ RT reagent Kit with gDNA Eraser (Perfect Real Time) (Code No. RR047A) and SYBR^®^ Premix Ex Taq™ II (Tli RNaseH Plus) (Code No. RR820A) were purchased from TaKaRa Biotechnology Co., Ltd. (Dalian, China).

### 2.2. Herbal Material and Preparation of TFs

*R. laevigata* Michx fruit was obtained from Yunnan Qiancaoyuan Pharmaceutical Company Co. Ltd. (Yunnan, China) and identified by Dr. Yunpeng Diao (College of Pharmacy, Dalian Medical University, Dalian, China). The crude extract was prepared and the content of TFs was 81.5% according to our previous work [22]. Briefly, the powder (500 g) of the *R. laevigata* Michx fruit was crushed and extracted with 60% aqueous ethanol (4 L) two times and at 2 h for each under heat reflux. The extracted solution was condensed under 60 °C and the produced residue was added into a D101 macroporous resin column. Then, in order to obtain the crude extract, the 40% ethanol fraction was collected and evaporated. Finally, according to the previous methods [27], the content of TFs in the crude extract was detected by colorimetric methods.

### 2.3. Animals

The TFs weresuspended in 0.5% sodium carboxyl methyl cellulose (CMC-Na). Male SD rats (180–220 g) were purchased from the Experimental Animal Center at Dalian Medical University (Dalian, China) (SCXK: 2013-0003). All experimental procedures were approved by the Animal Care and Use Committee of Dalian Medical University (approval number: SYXK (Liao) 2013-0108; 8 November 2013), and performed in strict accordance with the PR China Legislation Regarding the Use and Care of Laboratory Animals. The rats were allowed to adapt to the new environment for one week before the experiments, which were housed in a room under 12 h light/dark cycles, a relative humidity of 60% ± 10%, and a controlled temperature of 22 ± 3 °C. The rats were group housed and allowed ad libitum access to water and a standard pellet diet throughout the experiment.

### 2.4. Pharmacological Treatments and I/R

The rats were randomly divided into eight groups: animals (*n* = 32) in vehicle groups were treated with 0.5% CMC-Na; animals (*n* = 32) in TF groups were treated with TFs, which were administered intragastrically (i.g.) to the animals at the doses of 200 mg/kg once daily for seven consecutive days. On the eighth day, the model of 70% partial hepatic ischemia as described previously was performed [28]. Previous studies have implemented a time course to detect the optimal ischemia time period for inducing liver injury [29,30]. The results indicated that less than 60 min of ischemia produced only minimal transaminase elevations, whereas greater than 75 min of ischemia was poorly tolerated with gross evidence of poor reperfusion of the ischemic lobes. Therefore, a reproducible level of liver injury was observed using 1 h of ischemia and, thus, used for the modeling methods in this paper. In addition, the activities of AST and ALT were of greater relevance to the times of reperfusion. Thus, we carried out different times of reperfusion (2 h, 6 h, and 24 h). Briefly, the rats were anesthetized, and the livers were exposed by midline laparotomy, then the inflow of the left lateral and median lobes of the livers were choked by placement of a bulldog clamp, while the right lobes were remained perfused to prevent intestinal congestion occlusion. After 1 h of hepatic ischemia, the bulldog clamp was removed and the liver was reperfused by the blood. Furthermore, the animals in vehicle and TF groups were divided into four groups: the rats in the sham groups underwent similar surgical procedures without I/R; the rats in the I/R groups were subject to 2, 6, and 24 h reperfusion, respectively. At the end of surgery, blood samples of all rats were obtained via the abdominal vein under anaesthesia. The left lateral lobes of livers were obtained after perfusing with 4 °C phosphate-buffered saline (PBS) and then fixed in 4% paraformaldehyde for histological examination. The median lobes were stored at −80 °C for the other assays.

### 2.5. Biochemical Assay

The activities of serum AST, ALT, MPO, and LDH in each group were measured by using the commercial kits according to the manufacturer’s instructions.

### 2.6. Histopathological Examination

Formalin-fixed liver samples were embedded in paraffin and cut for 5-μm slices, and then stained with hematoxylin and eosin (HE) according to the manufacturer’s instructions. The staining images were acquired using a light microscope (Leica DM4000B, Solms, Germany) with 200× magnification.

### 2.7. Transmission Electron Microscopy (TEM) Assay

The liver tissue (<1 mm^3^) samples were harvested and fixed overnight at 4 °C in 2% glutaraldehyde. After washing in 0.1 M sodium cacodylate buffer, the samples were fixed in 1% osmium tetroxide for 2 h, and then dehydrated in gradient ethanol solutions. Finally, pretreated samples were used for ultramicrotomy and collected on copper grids. The obtained sections were then stained and observed using a transmission electron microscope (JEM-2000EX, JEDL, Tokyo, Japan).

### 2.8. Oxidative Stress Assay

The activities of MDA, SOD, and GSH in liver tissues were measured by using the commercial kits according to the manufacturer’s instructions.

### 2.9. Immunohistochemical Examination

Regarding the histopathological examination, the slices were incubated in 3% hydrogen peroxide (H_2_O_2_) for 30 min and normal goat serum to block nonspecific protein binding for 30 min. Then, the sections were incubated overnight at 4 °C with rabbit anti-Sirt1 or TLR4 antibody (1:100, dilution), followed by incubating biotin labeled goat anti-rabbit IgG and horseradish peroxidase-conjugated streptavidin for 15 min, respectively. Eventually, the slides were incubated in DAB solution for 10 min at 37 °C, counterstained by hematoxylin and mounted with neutral gum. Images were taken by a light microscope (Leica DM4000B, Solms, Germany) with 100× magnification. The optical density (IOD) of photographs were assayed by using Image-Pro Plus 6.0 (Media Cybernetics, Rockville, MD, USA).

### 2.10. Quantitative Real-Time PCR Assay

The total RNA samples were extracted by using RNAiso Plus reagent following the manufacturer’s protocol. The purity of the extracted RNA was determined, then reverse transcription polymerase chain reaction (RT-PCR) was performed using a PrimeScript^®^ RT reagent Kit following the manufacturer’s instructions with a TC-512 PCR system (TECHNE, Staffordshire, UK). The levels of mRNA expression were quantified by real-time PCR with SYBR^®^ PremixEx Taq™ II (Tli RNaseH Plus) and ABI 7500 Real-Time PCR System (Applied Biosystems, Waltham, MA, USA). The sequences of the primers for rats are shown in Table 1. A no-template control was analyzed in parallel for each gene, and the GAPDH gene was selected as the house-keeping gene in our study. Finally, the unknown template was calculated through the standard curve for quantitative analysis.

### 2.11. Western Blot Assay

Then, total protein, nuclear, and cytolymph proteins were extracted from the tissues using appropriate cold lysis buffer containing 1 mM phenylmethylsulfonyl fluoride (PMSF) based on the manufacturer’s instructions. Samples were loaded onto the SDS-PAGE gel (10%–15%), separated electrophoretically, and transferred onto a PVDF membrane (Merck Millipore, Merck KGaA, Darmstadt, Germany). After blocking non-specific binding sites for 3 h with 5% dried skim milk in TTBS at room temperature, the membrane was individually incubated overnight at 4 °C with primary antibodies (Table 2). Then the membrane was incubated at room temperature for 2 h with horseradish peroxidase-conjugated antibodies at a 1:5000 dilution. Protein expression was detected by an enhanced chemiluminescence (ECL) method and imaged using ChemiDoc XRS (BIO-RAD, Hercules, CA, USA). To eliminate the variations of protein expression, the data were adjusted to correspond internal reference expression (IOD value of target protein versus IOD of correspond internal reference).

### 2.12. Statistical Analysis

All of the data were analyzed using statistical software SPSS 18.0 (IBM, Almon grams, NY, USA) and expressed as means ± SD. Differences among groups were determined using one-way ANOVA, followed by a post hoc least-significant difference (LSD) test. Comparisons between the two groups were performed using an unpaired Student’s *t*-test. *p* < 0.05 and *p* < 0.01 were considered to be significant.

## 3. Results

### 3.1. TFs Reduces the Levels of ALT, AST, MPO, and LDH after I/R Injury

**Figure 1:**
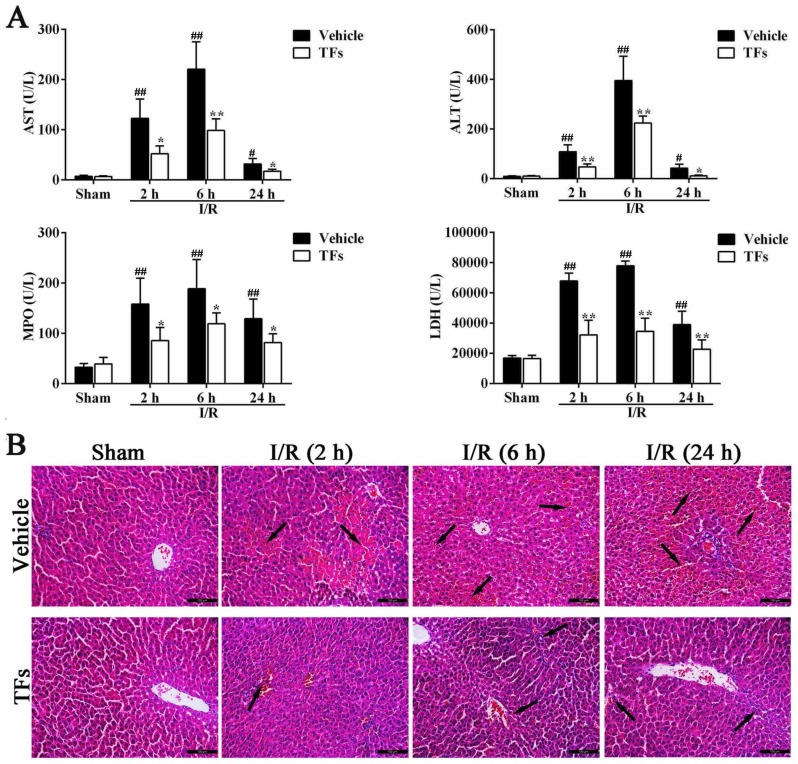
TFs reduced AST, ALT, MPO, and LDH activities after I/R injury. (**A**) Effects of TFs on serum AST, ALT, MPO, and LDH activities after 1 h of ischemia and different times of reperfusion (2 h, 6 h, and 24 h). Data are presented as the mean ± SD (*n* = 6). ^#^ *p* < 0.05 and ^##^ *p* < 0.01 versus sham; * *p* < 0.05 and ** *p* < 0.01 versus vehicle; and (**B**) effects of TFs on HE staining (200× magnification) after 1 h of ischemia and different times of reperfusion (2 h, 6 h, and 24 h).

As shown in Figure 1A, compared to the sham group, severe hepatotoxicity occurred and was quantified by the distinctly increased serum AST activities after 1 h of ischemia and different times of reperfusion (2 h, 6 h, and 24 h) with *p*-values of 0.003, 0.004, and 0.019, respectively. Similar results occurred in the serum ALT levels (*p*-values = 0.001, 1.97 × 10^−4^, and 0.023, respectively). However, pretreatment with 200 mg/kg of TFs markedly attenuated AST (*p*-values = 0.026, 0.002, and 0.046) and ALT (*p*-values = 0.036, 0.009, and 0.021) activities compared with vehicle groups after 2 h, 6 h, and 24 h reperfusion, respectively. In addition, compared to the sham group, 1 h of ischemia and different times of reperfusion (2 h, 6 h, and 24 h) significantly increased MPO (*p*-values = 0.005, 0.004, and 0.009) and LDH (*p*-values = 1.86 × 10^−8^, 2.43 × 10^−11^, and 0.002) levels in serum, respectively. However, TFs could markedly decrease MPO (*p*-values = 0.022, 0.048, and 0.044) and LDH (*p*-values = 3.61 × 10^−4^, 1.88 × 10^−5^, and 0.008) activities compared with the vehicle rats at 2 h, 6 h, and 24 h reperfusion, respectively.

### 3.2. TFs Attenuates I/R-Induced Liver Morphological Changes in Rats

As shown in Figure 1B, H and E staining results indicated that the rats in the model group showed obviously-increased areas of necrotic and inflammatory cell infiltration (the black arrow), correlating with significantly worsened hepatic functions compared with the vehicle group. In addition, there was sparing of the periportal area with progressively increased injury approaching the central vein. However, TFs (200 mg/kg) attenuated the I/R-induced morphological variations after 2 h, 6 h, and 24 h reperfusion.

### 3.3. TFs Improves I/R-Induced Cellular Structure Changes in Rats

**Figure 2:**
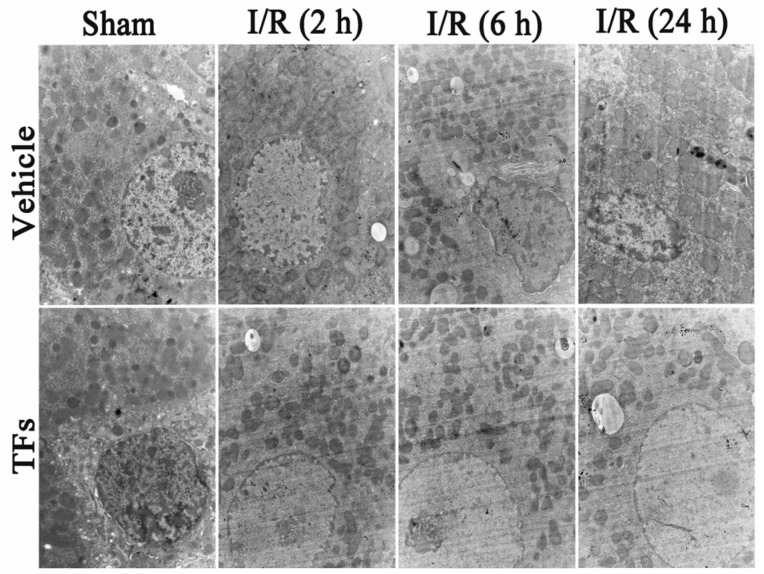
TFs improved I/R-induced cellular structure changes in rats. Effects of TFs on the ultrastructure (15,000× magnification) of hepatic cells after 1 h of ischemia and different times of reperfusion (2 h, 6 h, and 24 h).

As shown in Figure 2, the ultrastructure of hepatic cells was observed by TEM (15,000× magnification). The cell in I/R groups displayed nucleus chromatin condensation and marginalization, mitochondrial cristae break-down, and swelling after 2 h, 6 h, and 24 h reperfusion. However, TFs (200 mg/kg) improved I/R-induced cellular structure changes in rats.

### 3.4. TFs Improves I/R-Induced Oxidative Stress

**Figure 3:**
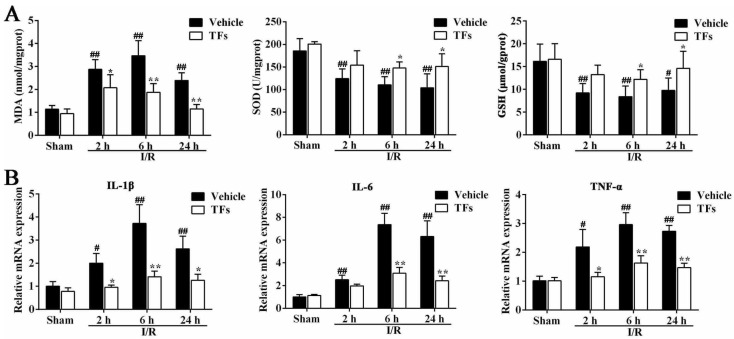
TFs inhibited I/R-induced oxidative stress and inflammation after I/R injury. (**A**) Effects of TFs on MDA, SOD, and GSH activities in liver tissue after 1 h of ischemia and different times of reperfusion (2 h, 6 h, and 24 h); and (**B**) effects of TFs on the mRNA levels of IL-1β, IL-6, and TNF-α in liver tissue after 1 h of ischemia and different times of reperfusion (2 h, 6 h, and 24 h). Data are presented as the mean ± SD (*n* = 6). ^#^ *p* < 0.05 and ^##^ *p* < 0.01 versus sham; * *p* < 0.05 and ** *p* < 0.01 versus vehicle.

As shown in Figure 3A, in I/R-treated group, the levels of MDA were increased compared with sham rats after 2 h (*p*-value = 5.04 × 10^−5^), 6 h (*p*-value = 1.57 × 10^−4^), and 24 h (*p*-value = 0.001) reperfusion. However, TFs significantly decreased the MDA levels (*p*-values = 0.046, 0.006, and 1.39 × 10^−5^) compared with the vehicle group after 2 h, 6 h, and 24 h reperfusion, respectively. In addition, the decreased levels of SOD (*p*-values = 0.002, 0.001, and 0.004) and GSH (*p*-values = 0.009, 0.004, and 0.021) were observed in I/R rats compared with sham group after 2 h, 6 h, and 24 h reperfusion, respectively. However, TFs (200 mg/kg) markedly decreased SOD (*p*-values = 0.151, 0.041, and 0.027) and GSH (*p*-values = 0.093, 0.049, and 0.029) levels after 2 h, 6 h, and 24 h reperfusion, respectively.

### 3.5. TFs Inhibits Liver Inflammation after I/R Injury

As shown in Figure 3B, in I/R-treated group, the mRNA levels of IL-1β (*p*-values = 0.020, 0.005, and 0.009), IL-6 (*p*-values = 0.004, 4.20 × 10^−4^ and 0.003) and TNF-α (*p*-values = 0.008, 0.002 and 3.03 × 10^−4^) were significantly increased compared with sham rats after 2 h, 6 h, and 24 h reperfusion, respectively, which were significantly downregulated by TFs.

### 3.6. TFs Downregulates SIRT1 and Upregulates TLR4 Protein Levels after I/R Injury

**Figure 4:**
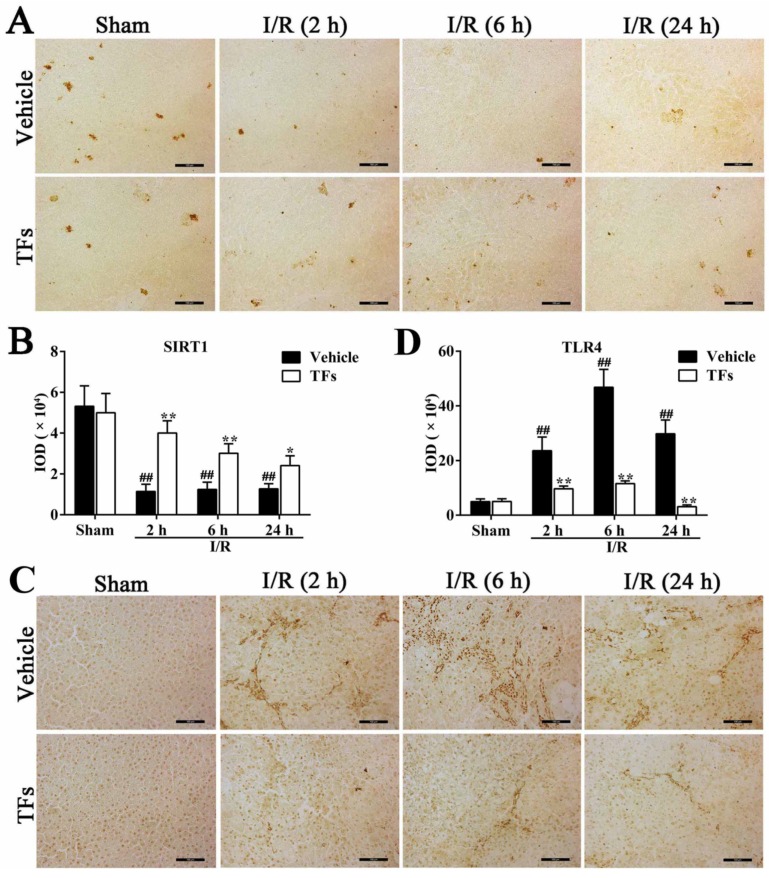
TFs downregulated Sirt1 and upregulated TLR4 protein levels after I/R injury. (**A**) Effects of TFs on Sitr1 protein level (brown areas) in liver tissue after 1 h of ischemia and different times of reperfusion (2 h, 6 h, and 24 h); (**B**) statistical analysis of the IOD values of Sitr1 protein level; (**C**) effects of TFs on TLR4 protein level (brown areas) in liver tissue after 1 h of ischemia and different times of reperfusion (2 h, 6 h, and 24 h); and (**D**) statistical analysis of the IOD values of TLR4 protein levels. Data are presented as the mean ± SD (*n* = 6). ^#^ *p* < 0.05 and ^##^ *p* < 0.01 versus sham; * *p* < 0.05 and ** *p* < 0.01 versus vehicle.

As shown in Figure 4A,B, fewer Sirt1-positive areas (brown areas) and decreased IOD values (*p* = 0.002, 0.003, and 0.002, respectively) were observed in I/R group compared with sham group after 2 h, 6 h, and 24 h reperfusion. However, compared to the vehicle group, TFs markedly increased Sirt1 protein levels (*p*-values = 0.002, 0.006, and 0.022, respectively) after 2 h, 6 h, and 24 h reperfusion. Immunohistochemical analysis also revealed that the protein levels of TLR4 (brown areas) and IOD values (*p*-values = 0.003, 3.98 × 10^−4^, and 0.001, respectively) were considerably increased in the I/R group, which were also significantly decreased by TFs (*p*-values = 0.009, 0.001, and 0.001, respectively) compared with vehicle group after 2 h, 6 h, and 24 h reperfusion (Figure 4C,D).

### 3.7. TFs Activate SIRT1/Nrf2-Mediated Signaling Pathway

**Figure 5:**
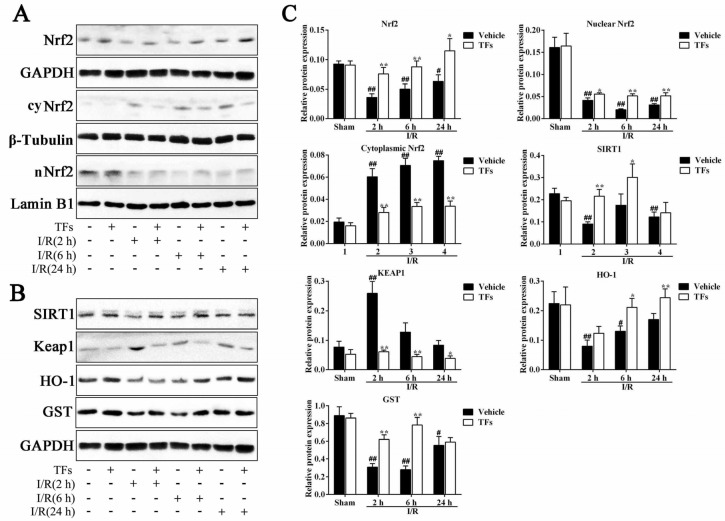
TFs activated the Sirt1/Nrf2-mediated signaling pathway. (**A**) Effects of TFs on Nrf2, nNrf2 (nucleus Nrf2), and cyNrf2 (cytoplasm Nrf2) proteins expression in liver tissue after 1 h of ischemia and different times of reperfusion (2 h, 6 h, and 24 h); (**B**) effects of TFs on Sirt1, KEAP1, HO-1, and GSH protein expression in liver tissue after 1 h of ischemia and different times of reperfusion (2 h, 6 h, and 24 h); and (**C**) statistical analysis of the Western blot assay. Data are presented as the mean ± SD (*n* = 6). ^#^ *p* < 0.05 and ^##^ *p* < 0.01 versus sham; * *p* < 0.05 and ** *p* < 0.01 versus vehicle.

As shown in Figure 5, in I/R-treated group, the total Nrf2 (*p*-values = 2.57 × 10^−4^, 0.002, and 0.015, respectively) and nuclear Nrf2 (nNrf2, *p*-values = 0.009, 0.009, and 0.009, respectively) levels were downregulated, and cytoplasmic Nrf2 (cyNrf2, *p*-values = 0.001, 2.77 × 10^−4^, and 5.84 × 10^−5^, respectively) levels were upregulated compared with sham rats after 2 h, 6 h, and 24 h reperfusion. However, compared to vehicle group, TFs significantly increased the total Nrf2 (*p*-values = 0.005, 0.007, and 0.020) and nNrf2 (*p*-values = 0.018, 4.35 × 10^−4^, and 0.008) levels, and decreased cyNrf2 level (*p*-values = 0.003, 0.001, and 3.25 × 10^−4^) after 2 h, 6 h, and 24 h reperfusion, respectively. Furthermore, compared with sham rats, the protein levels of Sirt1 (*p*-values = 0.001, 0.189, and 0.006), Keap1 (*p*-values = 0.002, 0.072, and 0.684), HO-1 (*p*-values = 0.005, 0.021, and 0.108), and GST (*p-*values = 0.001, 0.001, and 0.015) were downregulated after 2 h, 6 h, and 24 h reperfusion. However, TFs at the dose of 200 mg/kg dramatically upregulated the levels of Sirt1 (*p*-values = 0.002, 0.049, 0.557), Keap1 (*p*-values = 0.001, 0.008, 0.013), HO-1 (*p*-values = 0.071, 0.016, and 0.009), and GST (*p*-values = 0.001, 0.001, and 0.601) compared with vehicle groups after 2 h, 6 h, and 24 h reperfusion, respectively. These findings showed that TFs increased the antioxidant enzyme activities via activating Sirt1/Nrf2 signals.

### 3.8. TFs Inhibits TLR4 Signaling Pathway after I/R Injury

**Figure 6:**
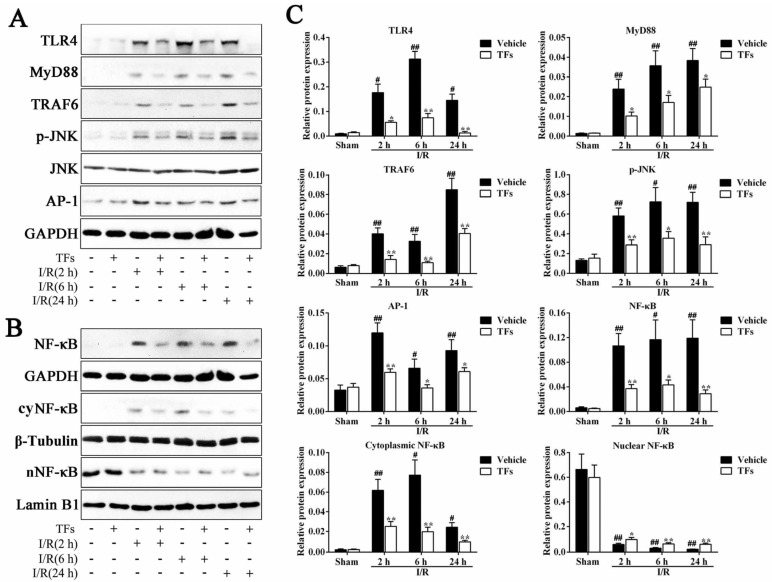
TFs inhibited the TLR4 signaling pathway after I/R injury. (**A**) Effects of TFs on TLR4, MyD88, TRAF6, p-JNK, and AP-1 protein expression in liver tissue after 1 h of ischemia and different times of reperfusion (2 h, 6 h, and 24 h); (**B**) effects of TFs on NF-κB, nNF-κB (nucleus NF-κB), and cyNF-κB (cytoplasm NF-κB) proteins expression in liver tissue after 1 h of ischemia and different times of reperfusion (2 h, 6 h, and 24 h); and (**C**) statistical analysis of the Western blot assay. Data are presented as the mean ± SD (*n* = 6). ^#^ *p* < 0.05 and ^##^ *p* < 0.01 versus sham; * *p* < 0.05 and ** *p* < 0.01 versus vehicle.

As shown in Figure 6, compared with sham rats, I/R significantly induced TLR4 levels (*p*-values = 0.014, 0.003, and 0.011) and suppressed the subsequent activation of its signaling effectors, reflected by the increased levels of MyD88 (*p*-values = 0.001, 0.001, and 4.39 × 10^−4^), TRAF6 (*p*-values = 0.001, 0.003, and 0.006), p-JNK (*p*-values = 0.001, 0.018 and 0.001), AP-1 (*p*-values = 0.001, 0.021, and 0.004) and NF-κB (*p*-values = 0.001, 0.027, and 0.003), respectively. However, 200 mg/kg TFs pretreatment notably decreased the protein levels of TLR4 (*p*-values = 0.023, 2.98 × 10^−4^, and 0.001), MyD88 (*p*-values = 0.012, 0.018, and 0.032), TRAF6 (*p*-values = 0.003, 0.006, and 0.004), p-JNK (*p*-values = 0.006, 0.016, and 0.004), AP-1 (*p*-values = 0.003, 0.022, and 0.034), and NF-κB (*p*-values = 0.005, 0.019, and 0.007) compared with vehicle groups after 2 h, 6 h, and 24 h reperfusion, respectively. Furthermore, after 2 h, 6 h, and 24 h reperfusion, the protein levels of cytoplasmic NF-κB (cyNF-κB, *p*-values = 0.001, 0.013, and 0.012) in ischemic liver were notably up-regulated, whereas nucleus NF-κB (nNF-κB, *p*-values = 0.001, 0.001, and 0.001) levels were markedly decreased. Compared with the vehicle group after 2 h, 6 h, and 24 h reperfusion, TFs obviously downregulated cyNF-κB (*p*-values = 0.006, 0.003, and 0.007), and upregulated nNF-κB (*p*-values = 0.026, 0.007, and 0.003) protein levels. The results also suggested that TFs inhibited the nuclear translocation from nucleus to cytoplasm of NF-κB in ischemic liver cells.

## 4. Discussion

Hepatic I/R injury, a frequent cause of liver failure, is related with liver transplantation, vascular surgery, and stroke [31,32]. A large number of studies have been carried out in the past several decades, but the pathogenesis of hepatic I/R injury has not been completely illuminated, and few medicines are available [33].

Previous studies have shown that liver reperfusion can increase cell injury by oxidative stress and inflammatory reactions [6]. Briefly, the early phase of hepatic I/R insult (within 2 h after reperfusion) involves the release of ROS and pro-inflammatory mediators [17]. The late phase (6–24 h after reperfusion) is featured with neutrophil-mediated inflammatory reaction [4]. ROS may result in lipid peroxidation, and activate signal transduction pathways, mitochondrial permeability transition, necrosis, and apoptosis of hepatocytes [8]. Larger amounts of complement factors, such as chemokines and cytokines, recruit neutrophils into the liver, which will insult hepatocytes through ROS release [3]. Therefore, the modulation of oxidative stress and inflammatory reactions represent promising therapeutic strategies to alleviate hepatic I/R injury.

TFs with potent anti-oxidative stress and anti-inflammatory actions have been shown in our previous research [24,34]. In the present work, a rat hepatic I/R model significantly increased serum AST, ALT, and LDH levels. However, pretreatment with TFs considerably reversed the alternations of these enzyme activities. The richest protein in neutrophils-MPO can be used as a quantitative measure of neutrophil infiltration [7]. Our results proved that TFs notably decreased neutrophil infiltration. In addition, HE staining results indicated that TFs exerted the protective action by decreasing coagulation necrosis with massive inflammatory cell infiltration in the liver. Furthermore, TEM assay results showed that TFs improved I/R-induced cellular structure changes in rats. Altogether, these results suggested that TFs have potent action for the prevention of hepatic I/R injury in rats.

High levels of SOD and GSH can protect hepatic I/R injury. SOD can catalytically reduce superoxide anion (O_2_^−^) to hydrogen peroxide, and GSH can catalyze the reduction of hydrogen peroxide [13]. MDA is an end-product of lipid hydroperoxide and an indicator of ROS [25]. The present paper indicated that SOD and GSH activities in the liver were markedly increased after TFs pretreatment compared with the model group, and MDA activity was dramatically decreased. Further results presented in this paper suggested that TFs significantly decreased the mRNA levels of IL-1β, IL-6, and TNF-α in the liver. These results proved the inhibition of oxidative stress and inflammatory response may be the potential mechanisms of TFs against hepatic I/R injury.

**Figure 7:**
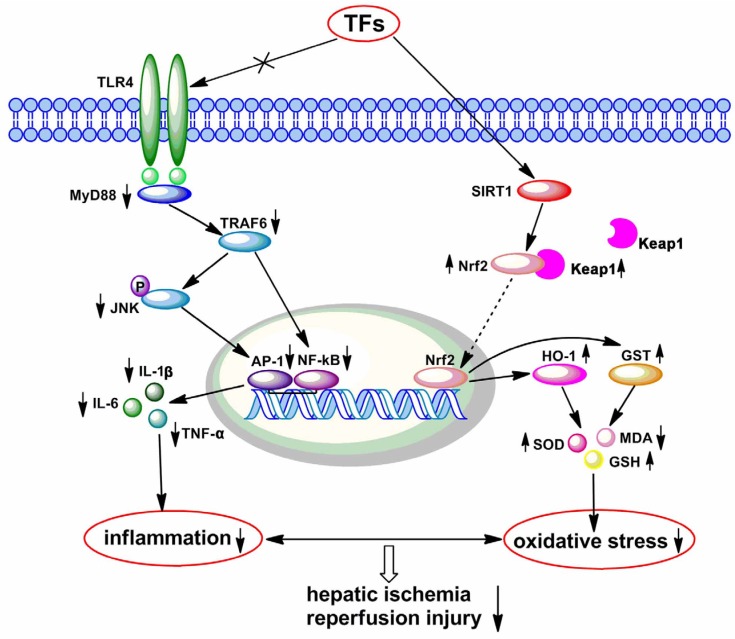
Proposed model for the protective effects of TFs against hepatic I/R injury. TFs alleviated liver I/R damage by regulating oxidative stress and inflammatory reactions through the inhibition of TLR4/MyD88 signaling and the activation of Sirt1/Nrf2 signaling.

A number of studies have shown that Sirt1 possesses a potent anti-oxidative effect, which can enhance transcriptional activity of Nrf2 [8]. Nrf2 plays a vital role in the inhibition of cellular oxidative stress by regulating intracellular redox homeostasis, which can also activate phase II antioxidants including HO-1 and GST [10]. Nrf2 can translocate from cytosol to nucleus when it is triggered, and lead to the increased antioxidant enzymes activities and decreased ROS induced insult [9,35]. In this paper, we found that TFs increased the levels of Sirt1, total Nrf2, nuclear Nrf2, HO-1, GST, and decreased cytoplasmic Nrf2 level in liver tissue. These results suggested that the anti-I/R effect of TFs might be through increasing the Sirt1 level and activating the Nrf2/ARE pathway (Figure 7).

The latest evidence suggests that TLR4 signaling plays a vital role in the progress of liver inflammation after I/R [36]. In detail, the activation of TLR4 signaling at the plasma membrane triggers NF-κB and AP-1 signaling, which are the vital regulators of some genes involved in inflammation [37]. Western blotting results in the present work proved that TFs downregulated TLR4 and downstream protein levels, including MyD88, TRAF6, p-JNK, NF-κB, and AP-1. In addition, TFs also inhibited the level and translocation of NF-κB. These findings indicated that the effects of TFs against hepatic I/R damage may be through inhibiting inflammation via adjusting TLR4 signaling (Figure 7).

Our previous studies have shown that the main chemicals of the product were flavonoids, with a content of 80.5% based on the chemical reactions and colorimetric method. The HPLC analysis results further proved that the contents of quercetin, kaempferide, and isorhamnetin in TFs were 3.11%, 2.72%, and 1.49%, respectively. These flavonoid constituents form in the pathophysiology, signaling, and the subsequent hepatic protection. However, other flavonoid substances in the crude extract were still unknown, and we will perform a deep investigation into the chemicals of TFs in our future work.

## 5. Conclusions

In summary, TFs have good protective effects against hepatic I/R injury by inhibiting oxidative stress and inflammation. Accordingly, TFs represent a novel and potent candidate for the treatment of I/R-induced liver injury in the future. Of course, further investigations are needed to deeply elucidate the mechanisms and clinical applications of the natural product.

## Figures and Tables

**Table 1:** The primer sequences used for real-time PCR assay in rats.

Gene	GenBank Accession	Full Name	Primer (5′–3′)
TNF-α	NM_012675.3	Tumour necrosis factor alpha	Forward: TCAGTTCCATGGCCCAGAC; Reverse: GTTGTCTTTGAGATCCATGCCATT
IL-1β	NM_031512.2	Interleukin-1 beta	Forward: CCCTGAACTCAACTGTGAAATAGCA; Reverse: CCCAAGTCAAGGGCTTGGAA
IL-6	NM_012589.1	Interleukin-6	Forward: ATTGTATGAACAGCGATGATGCAC; Reverse: CCAGGTAGAAACGGAACTCCAGA

**Table 2:** The information of the antibodies used in the present work.

Antibody	Full Name	Source	Dilutions	Company
Nrf2	Nuclear erythroid factor 2-related factorn2	Rabbit	1:1000	Proteintech Group, Chicago, IL, USA
Sirt1	Sirtuin 1	Rabbit	1:1000	Proteintech Group, Chicago, IL, USA
Keap1	Kelch-like ECH-associated protein 1	Rabbit	1:1000	Proteintech Group, Chicago, IL, USA
HO-1	Heme oxygenase-1	Rabbit	1:1000	Proteintech Group, Chicago, IL, USA
GST	Glutathione-S-transferase	Rabbit	1:1000	Proteintech Group, Chicago, IL, USA
TLR4	Toll like receptor 4	Rabbit	1:1000	Proteintech Group, Chicago, IL, USA
MyD88	Myeloid differentiation primary response gene (88)	Rabbit	1:1000	Abcam, Cambridge, UK
TRAF6	TNF receptor-associated factor 6	Rabbit	1:1000	Proteintech Group, Chicago, IL, USA
p-JNK	Phosphorylation of JNK	Rabbit	1:500	Bioworld Technology, San Luis, MN, USA
JNK	c-Jun *N*-terminal kinase	Rabbit	1:500	Bioworld Technology, San Luis, MN, USA
NF-κB	Nuclear factor kappa B	Rabbit	1:1000	Proteintech Group, Chicago, IL, USA
AP-1	Jun oncogene	Rabbit	1:1000	Proteintech Group, Chicago, IL, USA
β-Tubulin	Tubulin, beta	Rabbit	1:2000	Proteintech Group, Chicago, IL, USA
Lamin B1	Lamin B1	Rabbit	1:2000	Proteintech Group, Chicago, IL, USA
GAPDH	Glyceraldehyde-3-phosphate dehydrogenase	Rabbit	1:5000	Proteintech Group, Chicago, IL, USA
